# PURULENT PERICARDITIS WITH TAMPONADE DUE TO H. INFLUENZAE IN AN IMMUNE COMPETENT HOST

**DOI:** 10.51894/001c.122902

**Published:** 2024-08-30

**Authors:** Andrew Good, Mckalin Cox, Alyssa Wood, Christopher Nedzlek

**Affiliations:** 1 Emergency Medicine Henry Ford Wyandotte; 2 Emergency Medicine . Henry Ford Wyandotte

## 40

### INTRODUCTION

Since the development of vaccinations and antibiotics, purulent pericarditis has become a rare cause of visits to the Emergency Departments. With incidence of pericardial disease as low as 0.1% in hospitalized patients with chest pain, and variable management depending on etiology, the emergency Physician must recognize and treat rapidly and accurately. Risk factors for this life-threatening malady include immune-compromise, intrathoracic infection, and recent thoracic surgeries. *H. influenzae* is an uncommon organism to cause purulent pericarditis, with only 15 reported cases by 2006.

### CASE DESCRIPTION

We present a case of purulent pericarditis presenting in tamponade in an immune competent host. This 49-year-old male presented with chest pain, shortness of breath and a viral prodrome. A consolidation on chest-x-ray prompted empiric administration of Ceftriaxone and Doxycycline soon after presentation. He later became hypotensive, and bedside echocardiography noted tamponade physiology. Nearly 1 liter of purulent fluid was aspirated from his pericardial space with resultant clinical improvement and survival to discharge. The culture of this fluid grew *H. influenzae*, likely representing hematogenous spread from his pneumonia. The patient received two weeks of Ceftriaxone with resolution of his symptoms.

### DISCUSSION

Recognition of tamponade requires swift utilization of bedside echocardiography, with the expertise to recognize diastolic collapse of the right ventricle. Pericardiocentesis is best performed by a cardiologist, but in the setting of hemodynamic instability this falls to the emergency Physician. Broad spectrum antibiotic coverage should not be delayed, though gram-positive organisms are most often implicated. *H. influenzae* is an incredibly rare cause of purulent pericarditis with *Staph aureus* being most common in developed countries. If the patient has recently traveled, one must consider *M. tuberculosis* as this is the most common cause of purulent pericarditis worldwide and is frequently associated with coinfection with HIV.

